# Oral Health Status, Knowledge, Hygiene Behavior and Cognitive Function Among Older Adults in Shanghai, China

**DOI:** 10.3390/bs16081373

**Published:** 2026-08-10

**Authors:** Fangzhou Ye, Xiaomin Qu, Renhao Li, Yiran Wan, Jiangyao Zhu, Junwen Yu, Xiang Qi

**Affiliations:** 1 Rory Meyers College of Nursing, New York University, New York, NY 10010, USA; fy2285@nyu.edu (F.Y.); rl5235@nyu.edu (R.L.); yw7153@nyu.edu (Y.W.); 2 School of Social Development, East China University of Political Science and Law, Shanghai 201620, China; millie_qu@163.com; 3 Department of Epidemiology and Population Health, Albert Einstein College of Medicine, Bronx, NY 10461, USA; jiangyao.zhu@einsteinmed.edu; 4 NYU Shanghai, Shanghai 200124, China; jy5197@nyu.edu

**Keywords:** Alzheimer’s disease, dementia, dentistry, tooth loss, periodontitis, Chinese

## Abstract

Cognitive impairment is a growing concern in rapidly aging societies, including China, and oral health has been proposed as a modifiable correlate of cognitive aging; however, few studies compare distinct oral health domains within the same older population or identify the subgroups in which associations are strongest. We analyzed data from 1707 community-dwelling adults aged 55+ in the 2022 Lifelong Learning and Productive Aging Survey of Shanghai (LEAP-SH) study. Cognitive problems were measured with the Ascertain Dementia 8 (AD8) total score (0–8; higher = worse). Self-reported oral health, oral health knowledge, and oral hygiene behavior were each standardized and entered in separate models adjusted for sociodemographic and health covariates; interaction models tested effect modification by six sociodemographic moderators. All three domains were associated with lower AD8 scores, most strongly oral health status (β = −0.26; *p* < 0.001), followed by oral health knowledge (β = −0.14; *p* = 0.004) and oral hygiene behavior (β = −0.10; *p* = 0.036). Associations were broadly consistent across subgroups; only two interactions, both involving knowledge, were nominally significant, and neither survived correction for multiple comparisons. Oral health may be a modifiable correlate of cognitive aging, although the cross-sectional design precludes causal inference.

## 1. Introduction

Cognitive impairment is one of the most consequential health challenges of population aging. As populations age, cognitive decline and dementia are becoming more common, creating substantial burdens for affected individuals, families, and health systems (GBD 2019 Dementia Forecasting Collaborators, 2022; Jia et al., 2020). China is aging rapidly, and cognitive impairment is common among older Chinese adults (Qin et al., 2022; Ding et al., 2015). Shanghai offers a particularly informative setting for studying cognitive aging. By the end of 2024, 37.6% of its registered population was aged 60 or older, compared with roughly 22% nationally (Shanghai Municipal Office of the Working Commission on Aging et al., 2025), so the city entered population aging earlier and to a greater degree than the country overall, providing a preview of the aging profile that other large Chinese cities are projected to reach. Examining oral health and cognition in this population thus provides an early, policy-relevant view of associations likely to become increasingly salient across urban China, while Shanghai’s established community-health infrastructure supports high-quality data collection among older adults (Ma et al., 2025). Oral diseases, meanwhile, are among the most prevalent health conditions worldwide and remain a substantial and persistent burden in older populations (GBD 2021 Oral Disorders Collaborators, 2025) and in China specifically (Wang et al., 2025). Because options for treating established dementia remain limited, identifying modifiable factors associated with cognitive function is a public health priority (Livingston et al., 2024).

Oral health is a plausible and potentially modifiable correlate of cognitive function, and a growing body of longitudinal evidence has examined links between periodontitis and tooth loss with cognitive decline and incident dementia (Asher et al., 2022; Dibello et al., 2024; Farsi et al., 2026; R. Liu et al., 2025; Y. Liu et al., 2026; Qi et al., 2021, 2026), although the evidence remains heterogeneous (Qi et al., 2021). Several pathways have been proposed. Chronic oral inflammation, particularly periodontal disease, has been hypothesized to contribute to systemic and neural inflammation, with periodontal pathogens such as *Porphyromonas gingivalis* implicated in neuroinflammatory mechanisms, though causal claims remain debated (Dominy et al., 2019; Olsen, 2021; Hajishengallis, 2022). Impaired dentition and oral pain may reduce chewing capacity and dietary quality, with downstream effects on nutrition relevant to brain health (Tian et al., 2026). Oral hygiene behaviors and oral health knowledge may also be relevant: large cohort studies report that better oral hygiene care, including more frequent toothbrushing, is associated with lower dementia risk (Yoo et al., 2023; Park et al., 2026; Zhu et al., 2025), and knowledge and hygiene practices may reflect broader health literacy and self-care capacity, although evidence linking oral health literacy to behaviors and outcomes is mixed (Firmino et al., 2018; Baskaradoss, 2018). Together, these mechanisms suggest that multiple, distinct aspects of oral health could be relevant to cognition.

On another note, prior studies often focus on a single oral health indicator, such as periodontal disease or tooth loss (Qi et al., 2021; Gao et al., 2024; Ye et al., 2024), and fewer studies compare oral health status, oral health knowledge, and oral hygiene behavior in Chinese older populations. These domains are related but conceptually distinct, and can be understood as sitting at different points on a common causal chain: oral health knowledge reflects what a person knows about oral care, oral hygiene behavior reflects what they routinely do, and oral health status reflects the accumulated condition of the mouth that results, in part, from that behavior over time (Firmino et al., 2018; Baskaradoss, 2018). Knowledge is thus the most distal of the three and status the most proximal to the biological pathways linking oral health to cognition. Because they occupy different positions on this chain rather than being interchangeable indicators of a single underlying construct, they may differ in how strongly they relate to cognition, and any difference in the size of their associations is expected to reflect this conceptual ordering rather than an artifact of how each was measured. A further open question is whether these associations are uniform across the older population or concentrated in particular subgroups (Xie et al., 2025); identifying more vulnerable groups would help direct oral health promotion to those most likely to benefit, and recent work indicates that the cognitive correlates of oral health can vary across populations (Luo et al., 2026).

To address these gaps, we examined whether better oral health status, greater oral health knowledge, and more frequent toothbrushing were each associated with fewer reported cognitive problems. We also compared the relative strength of these three domains using standardized regression coefficients. We hypothesized that all three domains would be associated with fewer reported cognitive problems (H1–H3) and that oral health status would show the strongest association (H4), because among the three domains it lies closest to the mechanisms described above: it captures the accumulated oral disease burden and symptoms that feed both the inflammatory and the chewing- and nutrition-related pathways, whereas oral health knowledge and hygiene behavior are conceptually more distal and are generally thought to relate to cognition largely through their association with oral health status. As a secondary exploratory aim, we examined whether each association was modified by sociodemographic characteristics.

## 2. Methods

### 2.1. Data Source and Study Design

This study used cross-sectional data from the 2022 Lifelong Education for Aging Productively in Shanghai (LEAP-SH) survey, a population-based survey of community-dwelling older adults aged 55 years and older in Shanghai (Ma et al., 2025). The survey used a multistage sampling design to select neighborhoods, community clusters, and households; one age-eligible resident was interviewed in each household. Trained interviewers conducted face-to-face interviews and brief health assessments after informed consent was obtained. The analytic sample comprised 1707 participants. The sampling procedure has been described in detail elsewhere (Ma et al., 2025). The study was approved by the Research Ethics Committee, School of Social Development, East China University of Political Science and Law (Approval Number: ECUPL/SD-2223002), and all participants provided informed consent.

### 2.2. Outcome Measurement

Cognitive function was measured using the Ascertain Dementia 8 (AD8) total score (Galvin et al., 2005), derived from eight items assessing change in judgment, interest in activities, repeated questions, difficulty learning to use tools, confusion about the month or year, difficulty managing finances, remembering appointments, and persistent thinking or memory problems. Each item was recoded so that “yes, changed” = 1 and “no change” or “do not know” = 0. The eight items were summed to create the AD8 total score, ranging from 0 to 8, with higher scores indicating more cognitive problems. The AD8 is a brief screening instrument rather than a comprehensive neuropsychological assessment; the outcome analyzed here is the summed number of endorsed items (0–8), interpreted as the burden of reported cognitive problems on a screening measure. The AD8 has been validated for mild cognitive impairment and dementia screening among Chinese community-dwelling older adults (Wang et al., 2023).

### 2.3. Main Exposure Measurements

**Self-Reported Oral Health:** Oral health status was assessed by five items: a global self-rating of oral health together with four oral symptom items. In the global item, participants rated the overall condition of their mouth/teeth as excellent, very good, fairly good, fair, or poor. The four symptom items asked how frequently, over the past year, the participant had experienced (1) noticeable pain in the mouth; (2) difficulty eating certain foods because of problems with the teeth, mouth, or dentures; (3) sensitivity or discomfort of the teeth or gums to cold, hot, sour, or sweet foods; and (4) bleeding gums when brushing, each rated as always, fairly often, occasionally, almost never, or never (Qi et al., 2025a). The four symptom items were already scored so that higher values denoted fewer symptoms; the global self-rating was reverse-coded to match this direction (higher = better), and all five items were summed into a composite ranging from 5 to 25, with higher scores indicating better self-reported oral health.

**Oral Health Knowledge:** Oral health knowledge was measured with a score ranging from 0 to 8, based on eight items covering knowledge of toothbrush type, toothbrush replacement, brushing frequency, brushing duration, the importance of brushing for overall health, tongue cleaning, flossing frequency, and dental visit frequency. Each correct response was coded 1 and summed, with higher scores indicating greater knowledge, consistent with prior studies that operationalize oral health knowledge as the number of correct responses to items on toothbrushing, flossing, and dental care (Wong et al., 2024).

**Oral Hygiene Behavior:** Oral hygiene behavior was measured with a single item on habitual toothbrushing frequency. Participants were asked how often they brushed their teeth, in general, at the time of the survey; the item was not anchored to a specific recall period (e.g., the past six months, the past year, or lifetime) and therefore reflects current habitual practice. Responses were recorded on a six-level frequency scale: (1) rarely or never; (2) one to three times per month; (3) once per week; (4) two to six times per week; (5) once per day; (6) two or more times per day. Responses were used as scored, with higher values indicating more frequent toothbrushing. As a single self-reported item capturing current frequency, this measure does not reflect brushing technique, duration, or longer-term behavioral history. Toothbrushing frequency was chosen as the single oral hygiene indicator because toothbrushing is the most universal component of routine oral self-care and the behavior most consistently used as a single-item hygiene marker in population studies (Zhu et al., 2025; Yoo et al., 2023; Park et al., 2026). Although the survey also recorded flossing frequency, daily flossing was rare in this sample (13.1%) and showed negligible internal consistency with toothbrushing (Cronbach’s α = 0.09), indicating that the two behaviors capture distinct constructs and should not be combined into a single index. The limited evidence linking flossing to hard oral-health outcomes, particularly in older adults, further supported this choice (Worthington et al., 2019).

### 2.4. Covariates

All models were adjusted for sex, age, marital status, education, hukou type, annual personal income, number of chronic conditions, and BMI category. Sex was coded as male or female. Age was modeled as a continuous variable in years (range 55–85). Marital status was dichotomized as currently partnered or not partnered. Education was grouped into three levels: low (junior high school or below), medium (high school or technical/vocational school), and high (college or above). Hukou type was classified as agricultural or non-agricultural/other, based on the participant’s household-registration status at birth. Annual personal income was grouped into three categories: less than 40,000 RMB, 40,000–100,000 RMB, and 100,000 RMB or more. The number of chronic conditions was a count (range 0–8) of self-reported chronic conditions (comprising hypertension, diabetes, heart disease, stroke or other cerebrovascular disease, chronic bronchitis or other lung disease, arthritis, cancer, and other self-reported chronic conditions). Body mass index was categorized using cutoffs developed for Chinese adults—consistent with evidence that lower thresholds are appropriate for Asian populations (WHO Expert Consultation, 2004)—as normal or underweight (<24 kg/m^2^), overweight (24–27.9 kg/m^2^), or obese (≥28 kg/m^2^).

### 2.5. Statistical Analysis

We summarized sample characteristics using means and standard deviations (SD) for continuous variables and frequencies and percentages for categorical variables. The primary analysis used ordinary least squares (OLS) regression with the AD8 total score as the outcome. The AD8 total is a discrete, right-skewed count with a mass at zero and did not behave as an approximately continuous variable; we therefore did not rely on a normality assumption. OLS was retained because the estimand of interest, namely the covariate-adjusted mean difference in AD8 per one-SD increase in each standardized exposure, is well defined and directly interpretable irrespective of the outcome distribution. All models were estimated with heteroskedasticity-robust (Huber–White) standard errors (White, 1980). Three models were estimated: oral health status (Model 1), oral health knowledge with self-rated oral health (Model 2), and oral hygiene behavior with self-rated oral health (Model 3), each adjusted for the full covariate set. This structure reflects the conceptual ordering of the three domains.

Oral health status was entered as the five-item composite, whereas oral health knowledge and oral hygiene behavior are more distal and were each adjusted for self-rated oral health. To enable comparison of effect sizes across domains measured on different scales, the three main exposures were standardized to a mean of 0 and an SD of 1; the resulting coefficients represent the expected change in AD8 total score per one-SD increase in each predictor, and because all models share the same outcome and covariate set, the coefficients are directly comparable across domains. We assessed multicollinearity using pairwise correlations and variance inflation factors (VIFs). Self-rated oral health, entered as a covariate in Models 2 and 3, was only weakly correlated with oral health knowledge (r = 0.03) and oral hygiene behavior (r = 0.04). All VIFs were low (≤1.6; mean VIF ≈ 1.2), indicating no problematic collinearity. Because the global self-rating item is one component of the oral health status composite, self-rated oral health is included as a covariate only in Models 2 and 3 and never alongside the status composite, so their correlation does not affect estimation. We report coefficients (β) with 95% confidence intervals (CIs) and considered two-sided *p*-values below 0.05 as statistically significant.

As a sensitivity analysis, we re-estimated all three models using Poisson regression with robust standard errors. Within the analytic sample of 1707 participants, data were complete for the AD8 outcome, self-reported oral health status, oral hygiene behavior, and all covariates; missing values were limited to the oral health knowledge composite (6 participants, 0.4%). Models involving oral health status and oral hygiene behavior therefore included all 1707 participants, whereas models involving oral health knowledge included 1701. Given the small proportion of missing data and its restriction to a single measure, all models were estimated using complete-case analysis and no imputation was performed. Analyses were conducted in Stata 19.5 SE (StataCorp LLC, College Station, TX, USA).

### 2.6. Subgroup (Effect-Modification) Analyses

To explore whether associations differed across subgroups and to identify potentially vulnerable populations, we estimated interaction models for each of the three oral health exposures with six sociodemographic moderators: sex (male vs. female), marital status (currently partnered vs. not partnered), education (low [≤junior high] vs. medium [high school/technical] vs. high [college and above]), hukou type (agricultural vs. non-agricultural/other), income (<40,000 vs. 40,000–100,000 vs. ≥100,000 RMB), and age group (55–64 vs. 65–74 vs. 75–85 years). Each model added a product term between the standardized exposure and the moderator to the corresponding main-effects model, with the moderator removed from the covariate set to avoid duplication, yielding 18 interaction models. Effect modification was assessed with a joint Wald test of the product terms (the interaction *p*-value), and subgroup-specific associations were summarized as the marginal change in AD8 per one-SD increase in the exposure within each subgroup. These interaction models used the same specification as the primary analysis, so that subgroup estimates are directly comparable to the main results. Because 18 interaction tests were conducted, we interpreted individual results cautiously in light of multiple comparisons rather than relying on a single nominal threshold. Nominally significant interactions were treated as exploratory and hypothesis-generating.

## 3. Results

### 3.1. Sample Characteristics

**Figure A1:**
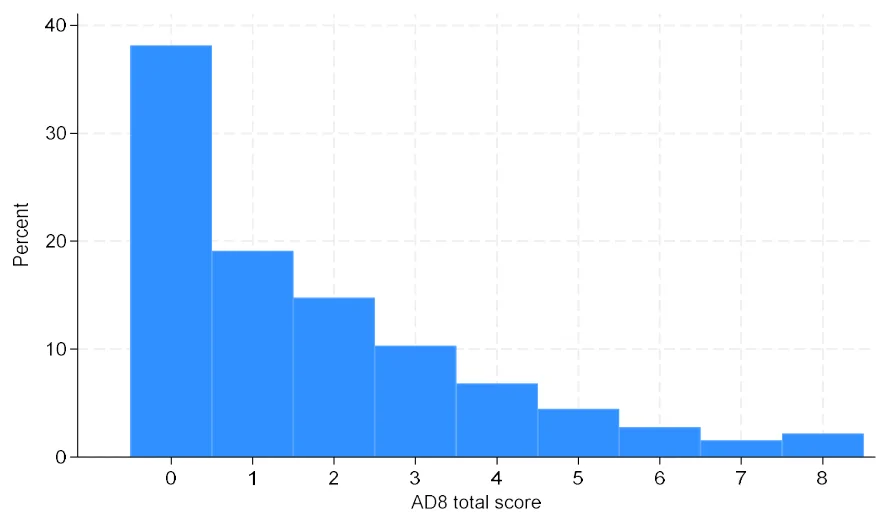
Distribution of AD8 total score among community-dwelling older adults, LEAP-SH 2022 (N = 1707).

Table 1 summarizes the characteristics of the 1707 participants. The mean age was 68.58 years (SD 6.65), and 58.3% were women. Most participants were currently partnered (82.8%), and slightly more than half had low educational attainment (≤junior high school; 51.6%). Non-agricultural hukou was held by 70.5% of participants, and most reported annual income between 40,000 and 100,000 RMB (64.7%). Participants reported a mean of 1.55 chronic conditions (SD 1.29), and 48.3% were overweight or obese. The mean AD8 total score was 1.74 (SD 2.01). The mean oral health score was 17.43 (SD 4.64), the mean oral health knowledge score was 4.62 (SD 1.31), and the mean toothbrushing frequency score was 5.71 (SD 0.74). The distribution of AD8 total scores among community-dwelling older adults is presented in Appendix A Figure A1.

### 3.2. Associations of Oral Health Domains with Cognitive Function

Table 2 presents the results from the three adjusted regression models. All three oral health domains were significantly associated with lower AD8 total scores after adjustment for sociodemographic and health-related covariates. Per one-SD increase, better oral health status was associated with a 0.26 point lower AD8 score (95% CI −0.35, −0.17; *p* < 0.001). After additionally adjusting for self-rated oral health, greater oral health knowledge was associated with a 0.14-point lower AD8 score (95% CI −0.23, −0.05; *p* = 0.004), and more frequent toothbrushing was associated with a 0.10-point lower AD8 score (95% CI −0.19, −0.01; *p* = 0.036). Self-rated oral health was independently associated with lower AD8 scores in both Model 2 and Model 3 (β = −0.08; *p* < 0.05). The three models explained a similar share of variance (R^2^ = 0.124, 0.116, and 0.113, respectively). Results were robust to modeling the AD8 count with Poisson regression: the associations for oral health status and knowledge remained significant and their relative ordering was preserved, whereas oral hygiene was attenuated to borderline significance (Appendix A Table A1).

### 3.3. Relative Strength Across Oral Health Domains

Because the three exposures were standardized, their coefficients are comparable across domains. Oral health status showed the largest standardized association with AD8 total score (β = −0.26), roughly twice the magnitude of oral health knowledge (β = −0.14) and more than twice that of oral hygiene behavior (β = −0.10). Oral health status also showed the most robust statistical evidence (*p* < 0.001) compared with knowledge (*p* = 0.004) and oral hygiene (*p* = 0.036).

### 3.4. Effect Modification by Sociodemographic Moderators

**Figure A2:**
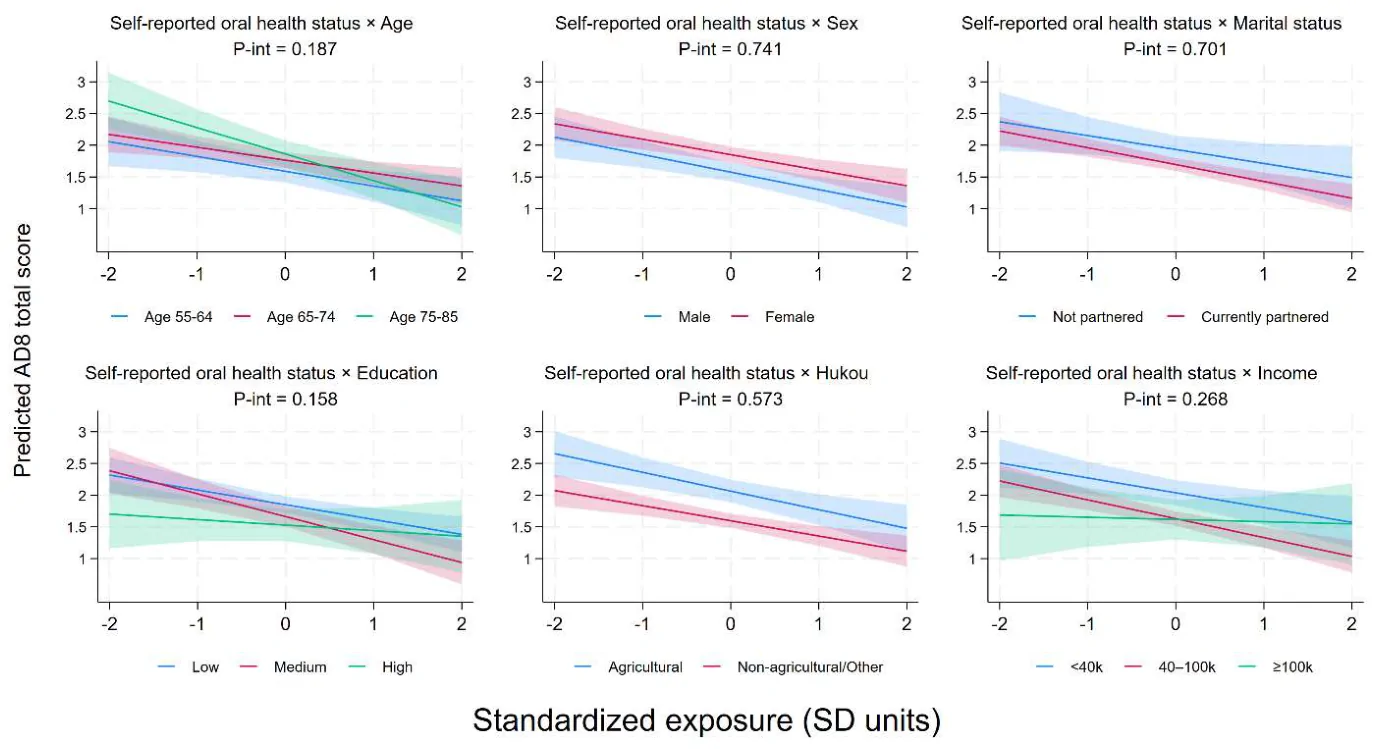
Effect modification of the association between self-reported oral health and cognitive function (AD8) across sociodemographic subgroups, LEAP-SH 2022 (N = 1707). Note. Each panel plots model-predicted AD8 total scores (lines) with 95% confidence intervals (shaded bands) for one sociodemographic moderator. Predictions are predictive margins from an ordinary least squares model containing the oral health status × moderator interaction and the full covariate set, averaged over the observed covariate distribution. The exposure is standardized (mean 0, SD 1) and plotted continuously from −2 to +2 SD (x-axis, SD units). P-int is the *p*-value for the joint exposure × moderator interaction contrast.

**Figure A3:**
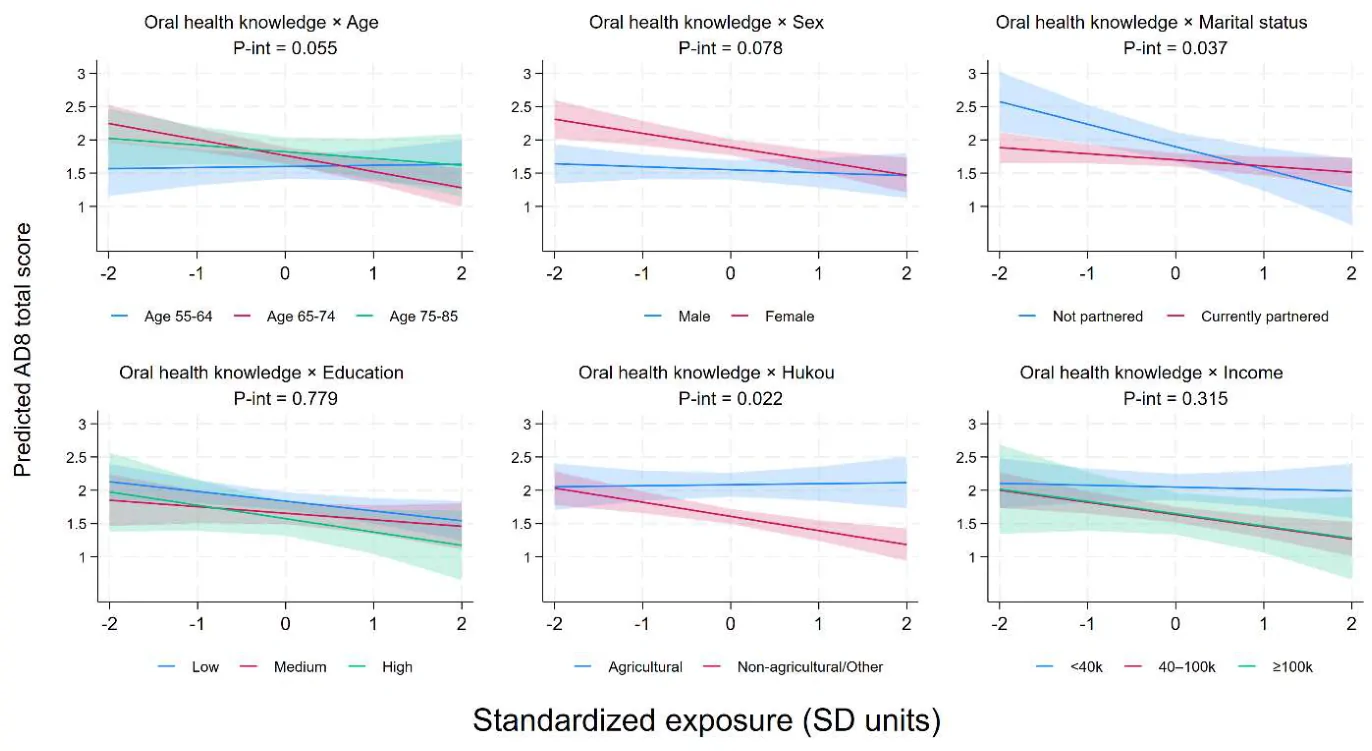
Effect modification of the association between oral health knowledge and cognitive function (AD8) across sociodemographic subgroups, LEAP-SH 2022 (N = 1701). Note. Each panel plots model-predicted AD8 total scores (lines) with 95% confidence intervals (shaded bands) for one sociodemographic moderator. Predictions are predictive margins from an ordinary least squares model containing the oral health knowledge × moderator interaction, self-rated oral health, and the full covariate set, averaged over the observed covariate distribution. The exposure is standardized (mean 0, SD 1) and plotted continuously from −2 to +2 SD (x-axis, SD units) rather than dichotomized into “low” and “high” categories; the moderator defines the separate lines within each panel. P-int is the *p*-value for the joint exposure × moderator interaction contrast.

**Figure A4:**
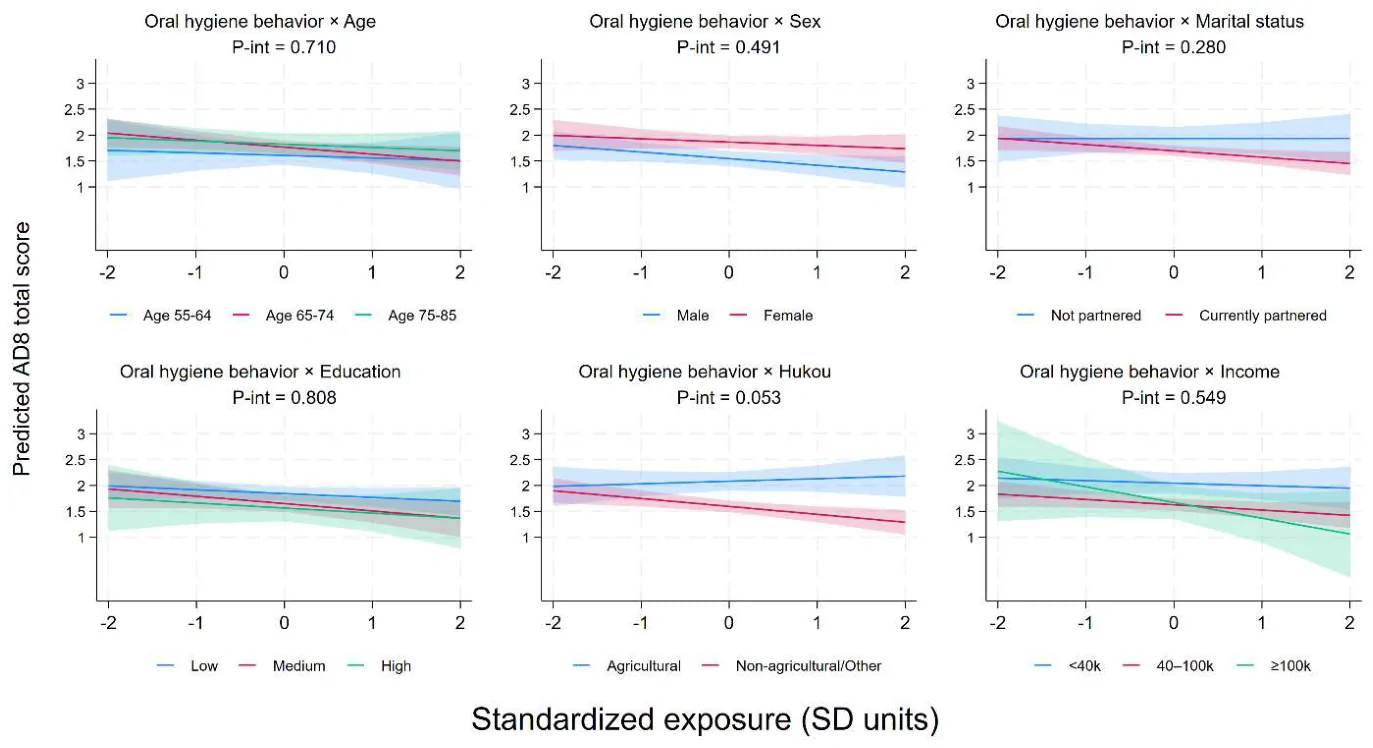
Effect modification of the association between oral hygiene behavior and cognitive function (AD8) across sociodemographic subgroups, LEAP-SH 2022 (N = 1707). Note. Each panel plots model-predicted AD8 total scores (lines) with 95% confidence intervals (shaded bands) for one sociodemographic moderator. Predictions are predictive margins from an ordinary least squares model containing the oral hygiene behavior × moderator interaction, self-rated oral health, and the full covariate set, averaged over the observed covariate distribution. The exposure is standardized (mean 0, SD 1) and plotted continuously from −2 to +2 SD (x-axis, SD units) rather than dichotomized into “low” and “high” categories; the moderator defines the separate lines within each panel. P-int is the *p*-value for the joint exposure × moderator interaction contrast.

We tested whether each of the three associations differed across the six sociodemographic moderators, yielding 18 interaction models (Appendix A Figure A2, Figure A3 and Figure A4; full interaction coefficients and *p*-values in Appendix A Table A2). The associations were broadly consistent across subgroups, and for oral health status, the strongest domain, no moderator reached significance (all interaction *p* ≥ 0.11). Only two of the 18 interactions were nominally significant, both involving oral health knowledge (with hukou, interaction *p* = 0.022; with marital status, interaction *p* = 0.037), and in each case the association was concentrated among urban-origin and unpartnered adults rather than among the disadvantaged groups hypothesized a priori. Because 18 tests were performed, roughly one nominal result is expected by chance, and neither survived Bonferroni correction (0.05/18 ≈ 0.003). This null pattern was robust to modeling the AD8 count with Poisson regression, which again produced two of 18 nominal interactions with none surviving correction and a different significant pair, consistent with chance findings.

## 4. Discussion

Across three distinct domains of oral health, better oral health status, greater knowledge, and more frequent toothbrushing were each associated with fewer cognitive problems, indicating that the link between oral health and cognition does not rest on any single aspect of oral health. The strongest and most consistent signal came from the condition of the mouth itself, the accumulated symptoms and the disease a person carries, rather than from what people knew or how often they brushed. These associations held broadly across sex, urban and rural hukou, education, and income, suggesting that oral health is a widely relevant factor in cognitive aging rather than a concern confined to any one group. Because oral health is potentially modifiable, it may be a meaningful target for supporting cognitive health, although the cross-sectional design cannot establish whether better oral health protects cognition, whether declining cognition erodes oral care, or both.

Several mechanisms may account for these associations. Oral health status reflects accumulated oral disease burden and symptoms, which lie closest to the biological pathways most often proposed to connect oral health and cognition: chronic periodontal inflammation and periodontal pathogens have been implicated in neuroinflammatory and systemic-inflammatory processes (Dominy et al., 2019; Olsen, 2021; Hajishengallis, 2022; Luo et al., 2022), and periodontal disease may be linked to cognition partly through accelerated biological aging (Qi et al., 2025b). Knowledge and hygiene behavior are more distal, relating to cognition largely through their contribution to oral health status and overlapping with broader health literacy and self-care capacity, for which the evidence is mixed (Firmino et al., 2018; Baskaradoss, 2018). Consistent with status being the most proximal domain, self-rated oral health remained independently associated with AD8 after adjustment for knowledge and behavior. Oral health knowledge may also partly reflect general health literacy accumulated across the life course: individuals with greater health literacy may better understand health information, navigate healthcare systems, and adopt preventive behaviors, and the educational, socioeconomic, and healthcare environments that shape health literacy may independently contribute to cognitive reserve (Chen et al., 2022). Our findings align with prior work linking poor oral health to worse cognitive outcomes (Asher et al., 2022; Dibello et al., 2024; Qi et al., 2021; Kelekar et al., 2025) and extend it by comparing three oral health domains within one population rather than examining periodontitis or tooth loss alone.

Notably, we found little evidence that the oral health–cognition association was concentrated in the disadvantaged groups hypothesized a priori. That the strongest association, for oral health status, was uniform across sociodemographic subgroups supports its interpretation as a broadly relevant correlate of cognitive aging rather than a group-specific pattern, and we treat the two nominally significant knowledge interactions as hypothesis-generating only. Independent of statistical significance, the higher burden of poor oral health among rural-origin and lower-education adults means that directing oral health resources toward these groups remains warranted on equity grounds.

Several limitations should be considered. First, the cross-sectional design precludes causal inference and cannot establish temporal order. Because the exposures were measured concurrently with the AD8, the associations may also reflect reverse or bidirectional pathways in which declining cognition reduces oral self-care and increases oral symptom burden; this concern may be greater for the behavior and knowledge domains than for accumulated oral damage (Delwel et al., 2018). Second, the key measures, including the AD8, oral health status, knowledge, and hygiene behavior, are self-reported and subject to recall and social-desirability biases; nonetheless, the AD8 is a validated screening instrument (Galvin et al., 2005; Wang et al., 2023), and self-reported oral health measures are widely used and informative in population research (Wu et al., 2011). In particular, oral hygiene behavior was captured by a single self-reported item (toothbrushing frequency); although this is the most established single-item hygiene marker, it does not reflect brushing technique or duration, and a fuller behavioral battery was not available. Third, although we adjusted for a range of covariates, residual confounding cannot be excluded. In a sensitivity analysis we additionally adjusted for depressive symptoms, dental-care utilization, and functional limitation simultaneously. All three oral health associations remained significant. Although the models adjusted for household income, more specific economic factors were not captured, including household wealth and assets and out-of-pocket dental costs; these could jointly shape oral health and cognition. Similarly, beyond the partial adjustment afforded by marital status, we did not measure social isolation, which is a recognized shared risk factor for both poorer cognitive function and poorer oral health in older adults. Residual confounding by these factors, as well as by nutritional status, physical activity, and dental-service accessibility (as distinct from utilization), therefore remains possible. Fourth, the sample is from a single, highly urbanized city, which may limit generalizability; however, Shanghai is an informative setting given its advanced population aging, and the findings can be tested in other contexts.

Integrating oral health assessment into health promotion programs for older adults is a plausible avenue to explore. Several specific, low-cost steps follow from our findings. First, because oral health status showed the strongest association and symptomatic conditions were most closely linked to cognition, a brief self-reported oral-symptom screen (for example, items on oral pain, difficulty eating, and bleeding gums) could be added to the health assessments already delivered in community daycare centers and neighborhood health services, and used as a prompt for dental referral. Second, where cognitive screening tools such as the AD8 are already administered, a short oral-health item set could be collected alongside them, allowing oral and cognitive concerns to be flagged together within existing workflows rather than through a separate program. Third, because the burden of poor oral health was higher among rural-origin and lower-education older adults, outreach and referral efforts could prioritize these groups on equity grounds. We emphasize that these are practical options to be evaluated rather than established interventions; whether integrating oral health care improves cognitive outcomes cannot be determined from cross-sectional data. Future research should use longitudinal and, where possible, clinically anchored measures of oral health to clarify directionality, examine mediating pathways such as inflammation and nutrition, and test whether interventions that improve oral health are associated with better cognitive trajectories.

## 5. Conclusions

Among older adults in Shanghai, oral health status, oral health knowledge, and oral hygiene behavior were all associated with AD8-assessed cognitive problems, with oral health status showing the largest association. This association was consistent across sociodemographic subgroups, and there was little evidence that any group was distinctly more vulnerable. Oral health may represent a potentially modifiable correlate of cognitive aging. Given the cross-sectional design, longitudinal studies are needed to confirm directionality and to assess whether improving oral health is associated with healthier cognitive trajectories in later life.

## Figures and Tables

**Table 1:** Sample characteristics of study participants (N = 1707).

Characteristic	Mean (SD) or n (%)
Oral health domains	
Self-reported oral health status	17.43 (4.64)
Oral health knowledge score	4.62 (1.31)
Oral hygiene behavior score	5.71 (0.74)
Cognitive health	
AD8 total score	1.74 (2.01)
Age	68.58 (6.65)
Sex	
Male	711 (41.7%)
Female	996 (58.3%)
Marital status	
Not partnered	294 (17.2%)
Currently partnered	1413 (82.8%)
Educational attainment	
Low (≤Junior high school)	880 (51.6%)
Medium (High school/technical school)	580 (34.0%)
High (College and above)	247 (14.5%)
Hukou type	
Agricultural	503 (29.5%)
Non-agricultural/other	1204 (70.5%)
Income level	
<40k RMB	439 (25.7%)
40k–100k RMB	1105 (64.7%)
≥100k RMB	163 (9.5%)
Number of chronic conditions	1.55 (1.29)
Body mass index category	
Normal or underweight	882 (51.7%)
Overweight	622 (36.4%)
Obese	203 (11.9%)

Note. Values are mean (SD) for continuous variables and n (%) for categorical variables; SD = standard deviation. Oral health measures: self-reported oral health status is a five-item composite (range 5–25), with higher scores indicating better oral health; oral health knowledge is the number of correct responses to eight knowledge items (range 0–8), higher = greater knowledge; oral hygiene behavior is toothbrushing frequency (range 1–6), higher = more frequent brushing. AD8 total score is the summed count of endorsed items (range 0–8), with higher scores indicating more reported cognitive problems. Number of chronic conditions is a count (range 0–8). Body mass index categories use China-specific cutoffs (normal or underweight < 24, overweight 24–27.9, obese ≥ 28 kg/m^2^).

**Table 2:** Associations between standardized oral health domains and cognitive problems.

Variable	Model 1: Self-Reported Oral Health	Model 2: Oral Health Knowledge	Model 3: Oral Hygiene Behavior
	β-coefficients (95% confidence intervals)		
Main independent variables			
Self-reported oral health	−0.257 *** (−0.349, −0.165)	—	—
Oral health knowledge	—	−0.139 ** (−0.232, −0.045)	—
Oral hygiene behavior	—	—	−0.098 * (−0.189, −0.006)
Covariates			
Self-rated oral health	—	−0.083 * (−0.157, −0.009)	−0.083 * (−0.157, −0.009)
Female (ref. Male)	0.274 ** (0.081, 0.468)	0.344 ** (0.146, 0.542)	0.321 ** (0.125, 0.518)
Age	0.018 * (0.003, 0.032)	0.013 ^†^ (−0.001, 0.028)	0.015 ^†^ (−0.000, 0.029)
Currently partnered (ref. Not partnered)	−0.235 ^†^ (−0.477, 0.008)	−0.220 ^†^ (−0.465, 0.024)	−0.234 ^†^ (−0.478, 0.010)
Medium education (ref. Low education)	−0.195 ^†^ (−0.407, 0.017)	−0.175 (−0.390, 0.039)	−0.195 ^†^ (−0.408, 0.019)
High education (ref. Low education)	−0.333 * (−0.626, −0.041)	−0.272 ^†^ (−0.568, 0.023)	−0.278 ^†^ (−0.572, 0.016)
Non-agricultural hukou (ref. Agricultural hukou)	−0.470 *** (−0.688, −0.252)	−0.460 *** (−0.680, −0.240)	−0.481 *** (−0.700, −0.261)
Annual income: 40,000–99,999 RMB (ref. < 40k RMB RMB)	−0.410 ** (−0.648, −0.173)	−0.405 ** (−0.643, −0.166)	−0.414 ** (−0.653, −0.175)
Annual income: ≥100,000 RMB (ref. < 40k RMB RMB)	−0.391 * (−0.773, −0.009)	−0.395 * (−0.780, −0.010)	−0.404 * (−0.789, −0.019)
Number of chronic conditions	0.279 *** (0.205, 0.353)	0.310 *** (0.236, 0.383)	0.311 *** (0.238, 0.384)
Overweight (ref. Normal or Underweight)	0.003 (−0.192, 0.198)	−0.019 (−0.216, 0.177)	−0.020 (−0.216, 0.176)
Obese (ref. Normal or Underweight)	−0.036 (−0.326, 0.254)	−0.092 (−0.384, 0.200)	−0.086 (−0.378, 0.205)
Constant	0.874 (−0.181, 1.929)	1.234 * (0.149, 2.319)	1.212 * (0.129, 2.295)
Observations	1707	1701	1707
R^2^	0.124	0.116	0.113

Note. Values are ordinary least squares regression coefficients (β) with 95% confidence intervals; the outcome is the AD8 total score (range 0–8, higher = more reported cognitive problems). Oral health exposures: self-reported oral health status is a five-item composite (range 5–25, higher = better oral health); oral health knowledge is the number of correct responses to eight items (range 0–8, higher = greater knowledge); oral hygiene behavior is toothbrushing frequency (range 1–6, higher = more frequent brushing). All three exposures were standardized (mean 0, SD 1), so each coefficient is the change in AD8 per one-SD increase in that exposure. ^†^ *p* < 0.10; * *p* < 0.05; ** *p* < 0.01; *** *p* < 0.001.

## Data Availability

The datasets used and/or analyzed during the current study are available from the corresponding author on reasonable request.

## Appendix A

**Table A1:** Associations between standardized oral health domains and cognitive function (AD8): Poisson regression sensitivity analysis.

Variable	Model 1: Self-Reported Oral Health	Model 2: Oral Health Knowledge	Model 3: Oral Hygiene Behavior
	IRR (95% confidence intervals)		
Main independent variables			
Self-reported oral health	0.869 *** (0.826, 0.914)	—	—
Oral health knowledge	—	0.927 ** (0.878, 0.979)	—
Oral hygiene behavior	—	—	0.953 ^†^ (0.905, 1.004)
Covariates			
Self-rated oral health	—	0.950 * (0.911, 0.992)	0.951 * (0.912, 0.993)
Female (ref. Male)	1.175 ** (1.046, 1.319)	1.225 ** (1.089, 1.379)	1.209 ** (1.075, 1.359)
Age	1.010 * (1.001, 1.018)	1.007 ^†^ (0.999, 1.016)	1.008 ^†^ (1.000, 1.016)
Currently partnered (ref. Not partnered)	0.891 ^†^ (0.778, 1.020)	0.903 (0.790, 1.033)	0.894 (0.780, 1.025)
Medium education (ref. Low education)	0.891 ^†^ (0.783, 1.014)	0.900 (0.791, 1.023)	0.890 ^†^ (0.782, 1.012)
High education (ref. Low education)	0.809 * (0.672, 0.975)	0.835 ^†^ (0.692, 1.006)	0.830 ^†^ (0.689, 1.001)
Non-agricultural hukou (ref. Agricultural hukou)	0.784 *** (0.691, 0.891)	0.787 *** (0.692, 0.894)	0.777 *** (0.684, 0.882)
Annual income: 40,000–99,999 RMB (ref. < 40k RMB)	0.820 ** (0.718, 0.937)	0.823 ** (0.719, 0.941)	0.818 ** (0.715, 0.935)
Annual income: ≥100,000 RMB (ref. < 40k RMB)	0.802 ^†^ (0.632, 1.018)	0.805 ^†^ (0.635, 1.021)	0.800 ^†^ (0.631, 1.015)
Number of chronic conditions	1.154 *** (1.110, 1.199)	1.173 *** (1.129, 1.219)	1.175 *** (1.131, 1.221)
Overweight (ref. Normal or Underweight)	1.010 (0.902, 1.132)	0.998 (0.890, 1.120)	0.998 (0.891, 1.119)
Obese (ref. Normal or Underweight)	0.997 (0.848, 1.171)	0.963 (0.821, 1.128)	0.965 (0.821, 1.133)
Constant	0.985 (0.539, 1.802)	1.198 (0.638, 2.247)	1.191 (0.636, 2.229)
Observations	1707	1701	1707
Pseudo R^2^	0.067	0.063	0.061

Note. The outcome is AD8 total score (0–8; higher = more cognitive problems). Values are incidence rate ratios (IRRs) from Poisson regression with robust (Huber–White) standard errors, with 95% confidence intervals in parentheses; an IRR below 1 indicates a lower expected AD8 count. All oral health domain scores were standardized (mean 0, SD 1), so IRRs for these predictors are per one-SD increase. Dashes indicate predictors not included in a given model. ^†^ *p* < 0.10; * *p* < 0.05; ** *p* < 0.01; *** *p* < 0.001.

**Table A2:** Effect modification of the association between three oral health domains and cognitive function (AD8) across sociodemographic subgroups, LEAP-SH 2022 (N = 1701).

	Self-Reported Oral Health	Oral Health Knowledge	Oral Hygiene Behavior
	β-coefficients (95% confidence intervals)		
Age categories	p-interaction = 0.187	p-interaction = 0.055	p-interaction = 0.710
Age 55–64	−0.233 (−0.404, −0.063)	0.016 (−0.156, 0.189)	−0.050 (−0.319, 0.219)
Age 65–74	−0.203 (−0.330, −0.076)	−0.241 (−0.369, −0.114)	−0.136 (−0.262, −0.010)
Age 75–85	−0.417 (−0.615, −0.220)	−0.102 (−0.305, 0.102)	−0.063 (−0.214, 0.088)
Sex	p-interaction = 0.741	p-interaction = 0.078	p-interaction = 0.491
Male	−0.276 (−0.419, −0.132)	−0.045 (−0.185, 0.095)	−0.128 (−0.254, −0.002)
Female	−0.244 (−0.362, −0.127)	−0.210 (−0.333, −0.088)	−0.064 (−0.196, 0.068)
Marital status	p-interaction = 0.701	p-interaction = 0.037	p-interaction = 0.28
Not partnered	−0.220 (−0.430, −0.009)	−0.338 (−0.548, −0.129)	0.001 (−0.200, 0.201)
Currently partnered	−0.265 (−0.367, −0.164)	−0.093 (−0.195, 0.010)	−0.123 (−0.225, −0.021)
Education	p-interaction = 0.158	p-interaction = 0.779	p-interaction = 0.808
Low	−0.234 (−0.357, −0.110)	−0.147 (−0.271, −0.022)	−0.075 (−0.194, 0.045)
Medium	−0.362 (−0.519, −0.204)	−0.098 (−0.262, 0.067)	−0.142 (−0.306, 0.022)
High	−0.087 (−0.336, 0.161)	−0.201 (−0.449, 0.048)	−0.098 (−0.376, 0.180)
Hukou	p-interaction = 0.573	p-interaction = 0.022	p-interaction = 0.053
Agricultural	−0.294 (−0.454, −0.135)	0.015 (−0.146, 0.177)	0.050 (−0.125, 0.225)
Non-agricultural/Other	−0.239 (−0.350, −0.128)	−0.212 (−0.325, −0.100)	−0.152 (−0.259, −0.045)
Income	p-interaction = 0.268	p-interaction = 0.315	p-interaction = 0.549
<40k	−0.234 (−0.405, −0.063)	−0.028 (−0.198, 0.142)	−0.049 (−0.227, 0.129)
40–100k	−0.297 (−0.412, −0.183)	−0.184 (−0.303, −0.065)	−0.102 (−0.212, 0.007)
≥100k	−0.035 (−0.339, 0.269)	−0.184 (−0.466, 0.098)	−0.303 (−0.726, 0.120)

**Table A3:** Associations between standardized oral health domains and cognitive function (AD8): sensitivity analysis with additional adjustment for depressive symptoms, dental-care utilization, and functional status.

Variable	Model 1: Self-Reported Oral Health	Model 2: Oral Health Knowledge	Model 3: Oral Hygiene Behavior
	β-coefficients (95% confidence intervals)		
Main independent variables			
Self-reported oral health status (composite)	−0.146 ** (−0.238, −0.055)	—	—
Oral health knowledge	—	−0.163 *** (−0.253, −0.074)	—
Oral hygiene behavior	—	—	−0.092 * (−0.180, −0.005)
Covariates			
Self-rated oral health (single item)	—	−0.038 (−0.110, 0.035)	−0.043 (−0.116, 0.029)
Female (ref. Male)	0.176 ^†^ (−0.010, 0.361)	0.239 * (0.050, 0.427)	0.205 * (0.017, 0.392)
Age	0.017 * (0.003, 0.031)	0.014 ^†^ (−0.001, 0.028)	0.015 * (0.001, 0.029)
Currently partnered (ref. Not partnered)	−0.035 (−0.269, 0.198)	−0.006 (−0.240, 0.228)	−0.029 (−0.263, 0.205)
Medium education (ref. Low education)	−0.166 (−0.368, 0.037)	−0.140 (−0.344, 0.064)	−0.166 (−0.369, 0.038)
High education (ref. Low education)	−0.318 * (−0.598, −0.037)	−0.265 ^†^ (−0.547, 0.017)	−0.290 * (−0.571, −0.009)
Non-agricultural hukou (ref. Agricultural hukou)	−0.417 *** (−0.626, −0.208)	−0.396 *** (−0.606, −0.187)	−0.422 *** (−0.631, −0.214)
Annual income: 40,000–99,999 RMB (ref. < 40k RMB)	−0.330 ** (−0.557, −0.103)	−0.310 ** (−0.537, −0.083)	−0.325 ** (−0.552, −0.097)
Annual income: ≥100,000 RMB (ref. < 40k RMB)	−0.301 (−0.667, 0.065)	−0.293 (−0.659, 0.074)	−0.304 (−0.671, 0.062)
Number of chronic conditions	0.187 *** (0.115, 0.258)	0.195 *** (0.124, 0.267)	0.200 *** (0.128, 0.271)
Overweight (ref. Normal or Underweight)	−0.002 (−0.188, 0.185)	−0.018 (−0.205, 0.169)	−0.013 (−0.200, 0.174)
Obese (ref. Normal or Underweight)	−0.031 (−0.308, 0.245)	−0.076 (−0.353, 0.201)	−0.063 (−0.340, 0.214)
Depressive symptoms: Mild (ref. No depression)	1.074 *** (0.816, 1.332)	1.137 *** (0.881, 1.393)	1.124 *** (0.868, 1.379)
Depressive symptoms: Severe (ref. No depression)	2.426 *** (1.965, 2.888)	2.532 *** (2.070, 2.994)	2.508 *** (2.049, 2.967)
Last dental visit: 1–3 years ago (ref. Within 12 months)	−0.126 (−0.375, 0.122)	−0.196 (−0.445, 0.052)	−0.155 (−0.403, 0.093)
Last dental visit: 3–5 years ago	−0.155 (−0.465, 0.154)	−0.217 (−0.527, 0.094)	−0.204 (−0.515, 0.106)
Last dental visit: >5 years ago	0.000 (−0.275, 0.275)	−0.128 (−0.403, 0.147)	−0.083 (−0.356, 0.190)
Last dental visit: Never	−0.102 (−0.347, 0.144)	−0.223 ^†^ (−0.471, 0.026)	−0.182 (−0.430, 0.066)
Functional status: Moderate dependence (ref. Severe dependence)	0.933 (−0.217, 2.083)	0.965 (−0.186, 2.116)	0.876 (−0.276, 2.027)
Functional status: Mild dependence	1.199 * (0.066, 2.333)	1.240 * (0.106, 2.373)	1.186 * (0.051, 2.321)
Functional status: Independent	0.542 (−0.539, 1.622)	0.579 (−0.502, 1.659)	0.523 (−0.559, 1.604)
Constant	0.113 (−1.437, 1.662)	0.317 (−1.243, 1.876)	0.348 (−1.212, 1.909)
Observations	1707	1701	1707
R^2^	0.208	0.210	0.207

Note. Sensitivity analysis re-estimating the three primary models (Table 2) with three additional covariates entered simultaneously: depressive symptoms, dental-care utilization, and functional status. The outcome is the AD8 total score (range 0–8; higher scores indicate more reported cognitive problems). Values are ordinary least squares coefficients with 95% confidence intervals; a negative coefficient means a higher predictor value is associated with fewer cognitive problems. Oral health exposures: self-reported oral health status is a five-item composite (range 5–25), higher = better oral health; oral health knowledge is the number of correct responses to eight knowledge items (range 0–8), higher = greater knowledge; oral hygiene behavior is toothbrushing frequency (ordinal, higher = more frequent brushing). All three were standardized (mean 0, SD 1) before modeling, so each coefficient is the change in AD8 per one-SD increase. ^†^ *p* < 0.10; * *p* < 0.05; ** *p* < 0.01; *** *p* < 0.001.
